# Amplified Mycobacterium Tuberculosis Direct Test for Diagnosing Tuberculous Pleurisy—A Diagnostic Accuracy Study

**DOI:** 10.1371/journal.pone.0044842

**Published:** 2012-09-10

**Authors:** Chieh-Mo Lin, Shu-Min Lin, Fu-Tsai Chung, Horng-Chyuan Lin, Kang-Yun Lee, Chien-Da Huang, Chih-Hsi Kuo, Chien-Ying Liu, Chun-Hua Wang, Han-Pin Kuo

**Affiliations:** 1 Division of Pulmonary and Critical Care Medicine, Chang Gung Memorial Hospital, Chiayi Branch, Chiayi, Taiwan; 2 Pulmonary Disease Research Center, Department of Thoracic Medicine, Chang Gung Memorial Hospital, Chang Gung University, School of Medicine, Taipei, Taiwan; McGill University, Canada

## Abstract

**Background:**

The study was designed to investigate the clinical usefulness of Amplified Mycobacterium Tuberculosis Direct (AMTD) tests for diagnosing TB pleurisy.

**Methods:**

One hundred and fifty-two patients for whom the exclusion of tuberculous pleural effusion was necessary were retrospectively analyzed.

**Results:**

The sensitivity of AMTD in diagnosing pleural TB was 36.4% (20 of 55). Combining sputum and pleural effusion AFB smear, pleural biopsy, and AMTD test of pleural effusion increased sensitivity to 82.5% (33/40). There were significantly higher percentages of neutrophils in the pleural effusion in the positive than in the negative AMTD group (38.0±6.7% vs. 11.1±3.7%, *p*<0.001). Patients with symptom duration <18 days prior to pleural effusion studies had more positive AMTD tests than those with symptom >18 days (70% vs. 31.4%; OR 5.09; 95% CI 1.54–16.79; *p* = 0.011).

**Conclusions:**

Combining AMTD tests with conventional diagnostic methods offer good sensitivity for pleural TB diagnosis. Patients in the early course of the disease are better candidates for AMTD tests.

## Introduction

Tuberculous (TB) pleural effusion is a common manifestation of extra-pulmonary TB and is the leading cause of pleural effusion in some geographical areas [1]–[3]. Pleural TB accounts for 4% of all TB cases in United States [4] and more than 10% in Spain [3]. Studies from Spain [3], Malaysia [2], and Saudi Arabia [2] show that TB accounts for 25%, 44%, and 37% of all pleural effusion, respectively. Rupture of the sub-pleural caseous focus in the lungs into the pleural space is considered the initial event in the pathogenesis of primary TB pleural effusion. The release of mycobacterial antigens into the pleural space is followed by a delayed hypersensitivity reaction [5]. This process may or may not involve viable bacilli entering the pleural space, and thus, the bacillary burden in the pleural space is usually low.

The diagnosis of TB pleurisy at an earlier stage is critical to its clinical outcome. It is a potentially curable disease if accurate diagnosis can be made and effective anti-tuberculosis treatment is administered early during the disease, as prognosis is worse if anti-tuberculous treatment is started more than 14 days after the initial visit [6]. Unfortunately, conventional diagnostic tests have limited clinical use. Microscopy of the pleural fluid for acid-fast bacilli (AFB) is positive in less than 5% of patients due to the pauci-bacillary nature of the disease [7], [8], whereas mycobacterial culture of the pleural fluid is time-consuming and has low sensitivity (24–58%) [8], [9]. Biopsy of pleural tissue for combined histologic examination and mycobacterial culture of the pleural fluid and tissue is the most sensitive of currently available diagnostic methods. This combination may lead to a diagnostic rate of 86% with conventional test and clinical diagnosis as reference standard [8]. Due to the limitation of each test, a combination of these tests have been recognized as the best approach for diagnosing TB pleurisy [7].

Because of the limitations of conventional tests, newer rapid tests, such as nucleic acid amplification tests (NAATs), have been evaluated. NAATs permit the amplification of a specific target RNA or DNA sequence that can be detected via a nucleic acid probe [10], [11]. Less than 10 organisms/mL may give a positive result [12], [13]. The Amplified Mycobacterium Tuberculosis Direct (AMTD) test, one of the commercial tests of NAATs, is approved by the US Food and Drug Administration (FDA) for use in smear-positive as well as smear-negative respiratory specimens. NAATs have excellent sensitivity and specificity (>95%) in AFB smear-positive respiratory specimens like sputum [14], but have variable sensitivities, ranging from 11% to 100% in diagnosing pleural TB [15]. Thus, there has been no consensus regarding the usefulness of NAATs for diagnosing TB pleurisy.

This study was designed to examine the clinical usefulness and accuracy of AMTD tests for diagnosing TB pleurisy. In addition, this study also aimed to define the characteristics of patients with positive pleural AMTD.

## Materials and Methods

### Study population

**Figure 1:**
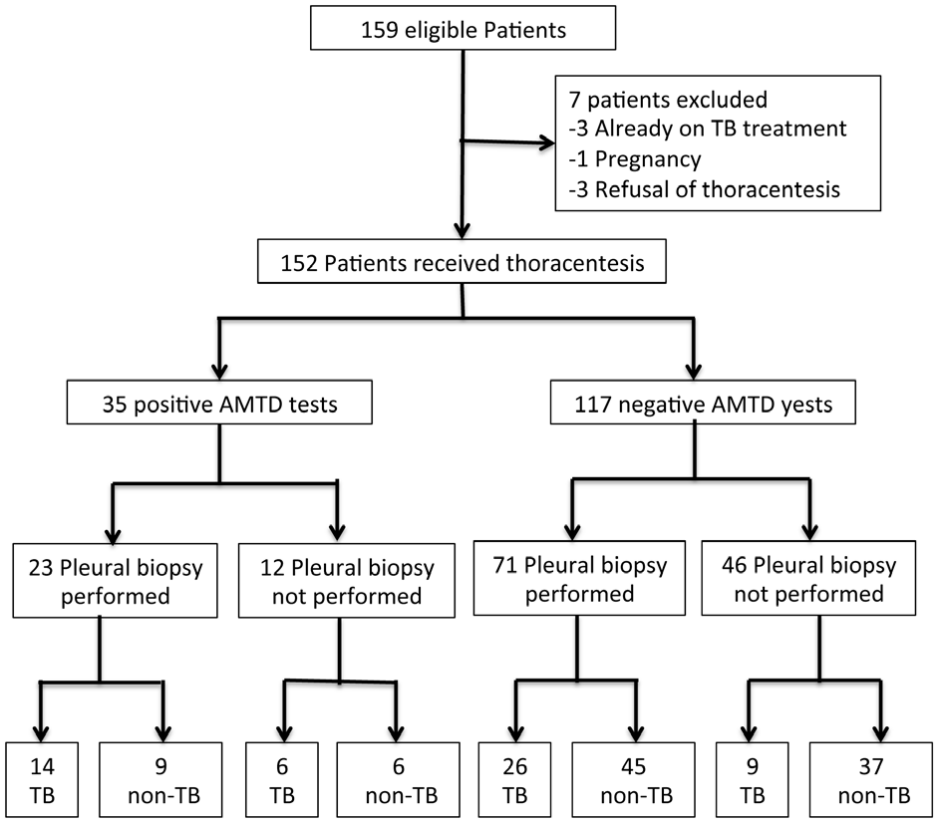
Patient flow chart for diagnosis of pleural effusion.

One hundred and fifty-nine consecutive patients were retrospectively analyzed from January 2005 to September 2010 at Linkou Chang-Gung Memorial Hospital, a university-affiliated hospital in Taiwan. The study recruited patients who had unilateral pleural effusion with clinical manifestations suggestive of tuberculosis, such as low-grade fever, cough, weight loss, or pleuritic pain. Seven patients were excluding due to already on TB treatment (n = 3), pregnancy (n = 1), and refusal of thoracentesis (n = 3). Thus, 152 patients received thoracentesis were entered for final analysis. For each patients, 50 ml of pleural fluid was collected and an aliquot of 10–15 ml pleural fluid submitted for cytology, routine cell count, AFB smear, *M. tuberculosis* culture, and biochemistry, including total protein, LDH, and sugar. The remaining 35–40 ml was used for AMTD test. Simultaneously, a pleural biopsy was performed and the specimens were submitted for histopathologic examination. But, there were 58 patients did not receive pleural biopsy due to inadequate amount of pleural effusion. The patient flow chart for diagnosis of pleural effusion was showed in figure 1. The Chang Gung Medical Foundation Institutional Review Board approved the study and waived the requirement for informed consent due to the retrospective nature of this study. Each patient's medical records were reviewed to collect the clinical characteristics and laboratory results. Respiratory symptoms were defined as cough, dyspnea, pleuritic chest pain, or hemoptysis. The duration of fever and symptom prior to medical study were collected.

### Diagnostic criteria of tuberculous pleurisy

The patients with TB pleurisy were defined according to a previous study, including both confirmed and probable TB pleurisy [15]. A patient with confirmed TB pleurisy was defined as having one of the following characteristics: pleural fluid sample that was culture-positive for *M. tuberculosis* and/or a histopathologic finding consistent with tuberculosis on pleural biopsy. A patient with probable TB pleurisy was defined as having one of the following characteristics: sputum specimen that was culture-positive for *M. tuberculosis* and/or other biologic specimens that were culture-positive for *M. tuberculosis*, and/or raised pleural lymphocytes and protein and/or a positive response to anti-tuberculosis medication without other possible causes of pleural effusion. According to previous studies [8], [9], patients with positive *M. tuberculosis* culture results represent a relatively small portion of all the cases of TB pleurisy. The study employed combination of pleural *M. tuberculosis* culture, pleural pathologic analysis, and clinical diagnosis as the reference method for diagnosing TB pleurisy. Total TB cases (confirmed + probable pleural TB cases) were used as a reference standard for diagnosis of TB pleurisy. AMTD test was not used for diagnosis of TB pleurisy. To reach the diagnosis of TB pleurisy, two senior physicians independently reviewed each patient's clinical and laboratory records and decided by consensus whenever there was a discrepancy (n = 1). In the study group, one heart failure patient had positive sputum *M. tuberculosis* cultures. The patient's pleural effusion was transudative nature and disappeared after diuretic therapy. After discussion by the 2 physicians, it was diagnosed as congestive heart failure induced pleural effusion rather than TB pleural effusion.

### Specimen processing

The pleural and sputum specimens were processed according to a previous report [16]. Briefly, pleural fluid and sputum specimens were stained with Ziehl-Neelsen stain for microscopic examination. Decontamination was performed using N-acetyl-L-cysteine-NaOH (BBL MycoPrep; Becton Dickinson, Cockeysville, MD, USA). The samples were cultured with BACTEC 12B vials (radiometric BACTEC 460TB system; Becton Dickinson, Franklin Lakes, NJ) and Lowenstein-Jensen media (Bio-Rad, Marnesla-Coquette, France). Nucleic acid amplification testing for *M. tuberculosis* complex was performed with the Gen-Probe AMTD assay according to the manufacturer's instructions [17].

The AMTD system amplified a specific rRNA target of *M. tuberculosis* via DNA intermediates and the amplified rRNA sequence (amplicon) was detected by hybridization with chemiluminescent acridinium ester-labelled probes. The specimen results were read in a Leader 450 luminometer. A cut-off value of 30,000 relative light units or more was used for diagnosing positive specimens. Positive and negative controls were included in every run. The AMTD test was interpreted without knowledge of the diagnosis of pleural effusion.

### Statistical analysis

Data were expressed as mean± SEM (standard error of the mean). The Student *t* test was used for comparison of continuous variables between different groups of tuberculous pleural effusion, while the Mann-Whitney test was used for non-normal distributions. Categorical variables were compared by **χ**2 or Fisher's exact test. Univariate logistic regression analysis with Odds ratios (OR) and 95% CI were used to assess the difference of diagnostic yields between the two groups. The sensitivity, specificity, positive and predictive values were expressed as 95% confidence intervals (95% CI). A *p*<0.05 was considered statistically significant. Analysis was carried out using the SPSS (version 13.0; SPSS; Chicago, IL) statistical software.

## Results

### Patient diagnosis

From January 2005 to September 2010, 55 of 152(36.2%) patients had TB pleurisy, including confirmed TB pleurisy in 30 and probable TB pleurisy in 25 patients (Table 1). Among the study population, none was HIV infected. The etiologies of non-tuberculous pleural effusion included malignant pleural effusion (n = 23, 15.1%), para-pneumonic effusion or empyema (n = 16, 10.5%), congestive heart failure (n = 4, 2.6%), connective tissue disease (n = 5, 3.3%), cirrhosis (n = 3, 2.0%), NTM (n = 2, 1.3%), and chronic renal failure (n = 1, 0.7%). The uninterpretable results in pathologic study for diagnosis of pleural TB were found in patients with inadequate amount or poor quality of pleural biopsy specimens and pathologic study showed non-specific granuloma. These cases were firstly analysed for criteria of probable TB pleurisy or non-TB diagnosis. But there were 43 patients whose etiology could not be defined. The pleural effusions were small to moderate amount and disappeared without anti-TB treatment or other specific therapy. These patients possibly had viral or partially treated bacterial infection. Their medical records were followed for 2–7 years and none of them developed tuberculosis. Therefore, they were categorized as non-TB cases of unknown etiology.

**Table 1:** The clinical diagnosis of patients with pleural effusion (n = 152).

Diagnosis	No (%)
Total TB cases (confirmed + probable pleural TB cases), n (%)	55 (36.2)
Confirmed TB case	30 (19.7%)
Probable TB case	25 (16.4%)
Malignant pleural effusion, n (%)	23 (15.1)
Para-pneumonic effusion/empyema	16 (10.5)
Congestive heart failure	4 (2.6)
Connective tissue disease	5 (3.3)
Cirrhosis	3 (2.0)
Non-tuberculous Mycobacterium	2 (1.3)
Chronic renal failure	1 (0.7)
Unknown etiology	43 (28.3)

Abbreviation: TB, tuberculosis.

### Sensitivity, specificity, and predictive values of diagnostic tools

Sensitivities, specificities, and predictive values of various diagnostic tools for TB pleurisy, including smear, culture, biopsy, and AMTD test, were shown in Table 2. These analyses were performed in confirmed pleural TB case, probable pleural TB case, and total pleural TB case (confirmed + probable pleural TB case). The sensitivities of sputum AFB smear and culture for *M. tuberculosis*, pleural effusion AFB smear and culture for *M. tuberculosis*, and AMTD testing of pleural effusion for the diagnosis of total pleural TB case were 12.7%, 29.1%, 7.3%, 30.9%, and 36.4%, respectively. Of the 55 patients with TB pleurisy, 40 underwent pleural biopsy, and the sensitivity of pleural biopsy was 50.0%.

**Table 2:** Sensitivity, specificity, and predictive values of smear, culture, biopsy and AMTD test for diagnosing tuberculous pleurisy.

	positive/tested	positive/tested	Sensitivity	Specificity	Positive predictive value	Negative predictive value
	TB	non-TB				
**Confirmed pleural TB cases**						
Sputum AFB smear	6/30	3/122	20.0(7.7–38.6)	97.5(93.0–99.5)	66.7(29.9–92.5)	83.2(76.1–88.9)
Sputum MTB culture	10/30	7/122	33.3(17.3–52.8)	94.3(88.5–99.7)	58.8(32.9–100)	85.2(78.1–90.7)
Pleural effusion AFB smear	4/30	0/122	13.3(3.8–30.7)	100(97.0–100)	100(39.8–100)	82.4(75.3–88.2)
Pleural effusion MTB culture	17/30	0/122	56.7(37.4–74.5)	100(97.0–100)	100(80.5–100)	90.4(84.1–94.8)
Pleural biopsy	20/22	0/72	90.9(70.8–98.9)	100(95.0–100)	100(83.2–100)	97.3(90.6–99.7)
Pleural effusion AMTD	6/30	29/122	20.0(7.7–38.6)	76.2(67.7–83.5)	17.1(6.6–33.7)	79.5(71.0–86.4)
**Probable pleural TB cases**						
Sputum AFB smear	1/25	2/97	4(0.1–20.4)	97.9(92.8–99.8)	33.3(0.8–90.6)	79.8(71.5–86.6)
Sputum MTBculture	6/25	1/97	24.0(9.4–45.1)	99.0(94.4–100)	85.7(42.1–99.6)	83.5(75.4–89.8)
Pleural effusion AFB smear	0/25	0/97	NA	NA	NA	NA
Pleural effusion MTB culture	0/25	0/97	NA	NA	NA	NA
Pleural biopsy	0/18	0/54	NA	NA	NA	NA
Pleural effusion AMTD	14/25	15/97	56.0(34.9–75.6)	84.5(75.8–91.1)	48.3(29.5–69.5)	88.2(79.8–94.0)
**Total pleural TB cases** (**confirmed + probable pleural TB cases**)						
Sputum AFB smear	7/55	2/97	12.7(5.3–24.5)	97.9(92.8–99.8)	77.8(40.0–97.2)	66.4(58.1–74.1)
Sputum MTB culture	16/55	1/97	29.1(17.6–42.9)	99.0(94.4–100)	94.1(71.3–99.9)	71.1(62.7–78.6)
Pleural effusion AFB smear	4/55	0/97	7.3(2.0–17.6)	100(96.3–100)	100(39.8–100)	65.5(57.3–73.2)
Pleural effusion MTB culture	17/55	0/97	30.9(19.1–44.8)	100(96.3–100)	100(80.5–100)	71.9(63.5–79.3)
Pleural biopsy	20/40	0/54	50.0(33.8–66.2)	100(93.4–100)	100(83.2–100)	73.0(61.4–82.7)
Pleural effusion AMTD	20/55	15/97	36.4(23.8–50.4)	84.5(75.8–91.1)	57.1(39.4–73.7)	70.1(63.9–78.2)
Sputum AFB+pleural effusion AFB + Pleural Biopsy	22/40	1/54	55.0(38.5–70.7)	98.2(90.1–100)	95.7(78.1–99.9)	74.7(62.9–84.2)
Sputum AFB+pleural effusion AFB+Pleural Biopsy +AMTD	33/40	14/54	82.5(67.2–92.6)	74.1(60.4–85.4)	70.2(55.1–82.7)	85.1(71.7–93.8)

Sensitivity, specificity, and predictive values are expressed as%(95% confidence interval) Abbreviations: AMTD, Amplified Mycobacterium Tuberculosis Direct; TB, tuberculosis; AFB, acid-fast bacilli; MTB, *M. tuberculosis.*

The pathologic results require 3–5 days after pleural biopsy. Compared with the time required for *M. tuberculosis* culture, pleural biopsy was categorized as a rapid diagnostic test. Regarding rapid diagnostic tests for TB pleurisy, the sensitivity of combined sputum and pleural effusion AFB smears, and pleural biopsy was 55.0% (22/40). In contrast, the combination of sputum and pleural effusion AFB smear, pleural biopsy, and AMTD test of pleural effusion had higher sensitivity of 82.5% (33/40).

### Clinical characteristics of patients with TB and non-TB pleural effusion

The clinical characteristics of patients with TB and non-TB pleural effusion were summarized in Table 3. In the TB group, there were significantly lower percentage of neutrophil count (p = 0.024) but higher percentage of lymphocyte count (p = 0.004) in the pleural effusion than those in the non-TB group. In the non-TB group, there were 2 patients had positive sputum AFB smear but their culture showed *M. avian complex*. Another heart failure patient had positive sputum *M. tuberculosis* cultures. The patient's pleural effusion was transudative nature and disappeared after diuretic therapy. Therefore, it was diagnosed as congestive heart failure induced pleural effusion rather than TB pleural effusion.

**Table 3:** Comparison between patients with tuberculous and non-tuberculous pleural effusion.

	Total patients (n = 152)	TB pleural effusion (n = 55)	Non-TB pleural effusion (n = 97)	*p* value
Age	65.7±1.5	65.0±2.6	66.0±1.8	.746
Male gender, n (%)	101 (66.4)	38 (69.1)	63 (64.9)	.721
Respiratory symptoms	137(90.1)	50(90.9)	87(89.7)	1.00
Cough	70(46.1)	29(52.7)	41(42.3)	.239
Presence of fever, n (%)	33 (19.7)	15 (27.3)	18(18.6)	.225
Duration of symptom prior to study, days	24.5±1.6	25.5±3.0	23.9±1.9	.634
Echography showing fibrin formation, n (%)	19 (12.5)	11 (20.0)	8(8.2)	.043
Pleural effusion WBC,/ µL	1403 ± 189.7	1282±198.1	1471±275.2	.634
% of neutrophil in pleural effusion	22.4 ± 2.2	15.9±2.9	26±2.9	.024
% of lymphocyte in pleural effusion	70.5 ± 2.4	77.5±3.6	66.5±3.1	.027
Pleural effusion total protein level, g/dL	4.2 ± 0.1	4.4±0.2	4.1±0.1	.156
Pleural effusion LDH level, U/L	312.1± 74.3	231.4±31.0	357.5±114.8	.417
Pleural effusion sugar level, mg/dL	124.8 ± 7.7	123.2±16.2	125.7±7.8	.872
Pleural effusion specific gravity	1.031 ± 0.001	1.033±0.001	1.030±0.001	.041
Positive sputum AFB smear, n (%)	9 (5.9)	7 (12.7)	2(2.1)	.011
Positive sputum M. tuberculosis culture, n (%)	17 (11.2)	16 (29.1)	1 (1.0)	<.0001
Positive pleural effusion AFB smear, n (%)	4 (2.6)	4 (7.3)	0(0)	.016
Positive pleural effusion M. tuberculosis culture	17 (11.2)	17 (30.9)	0(0)	<.0001
Positive pleural biopsy, n (%)	20/93 (21.5)	20/40 (50.0)	0/54 (0)	<.0001

Abbreviations: AMTD, Amplified Mycobacterium Tuberculosis Direct; WBC, white blood cell count; LDH, *lactate dehydrogenase;* AFB, Acid fast bacilli.

Respiratory symptoms were defined as cough, dyspnea, pleuritic chest pain, or hemoptysis.

### Clinical characteristics of TB pleurisy patients with positive and negative AMTD results

Of the 55 patients with TB pleurisy, 20 had positive AMTD tests and 35 had negative AMTD tests. The clinical characteristics of the 55 patients according to their respective AMTD test results were summarized in Table 4. Between the two groups, there was no significant difference in age of years, male gender, incidence of fever, pleural effusion WBC count, specific gravity, sugar, and LDH level. Among the various diagnostic tools for TB pleurisy, there was no significant difference in the diagnostic yields of sputum AFB smear and *M. tuberculosis* culture, and pleural effusion AFB smear and *M. tuberculosis* culture between the two groups. In the positive AMTD group, there was significantly higher percentage of neutrophil count in the pleural effusion than that in the negative AMTD group (38.0±6.7% vs. 11.1±3.7%, *p*<0.001). There was also lower lymphocyte count (56.5±6.3% vs. 83.8±4.6%, *p* = 0.001) and total protein level (4.0±0.2% vs. 4.7±0.2%, *p* = 0.036) in the pleural effusion of the positive AMTD group. In addition, patients with durations of symptoms prior to pleural effusion studies <18 days had higher positive rates of AMTD test than those with symptoms >18 days prior to pleural effusion study (70% vs. 31.4%; OR, 5.09; 95% CI, 1.54–16.79; *p* = 0.011).

**Table 4:** Comparison between patients with positive and negative AMTD in tuberculous pleural effusion specimens.

	Positive AMTD (n = 20)	Negative AMTD (n = 35)	*p* value
Age	64.3±4.8	66.2±2.9	.717
Male gender, n (%)	17 (85.0)	21 (60.0)	.072
Presence of fever, n (%)	6 (30.0)	9 (25.7)	.761
Duration of symptom prior to medical study ≤18 days	14(70%)	11(31.4%)	.011
Echography showing fibrin formation, n (%)	4 (20.0)	7 (20.0)	1.000
Pleural effusion WBC,/ µL	1405±450.3	1216±189.1	.653
% of neutrophil in pleural effusion	38.0±6.7	11.1±3.7	<.001
% of lymphocyte in pleural effusion	56.5±6.3	83.8±4.6	.001
Pleural effusion total protein level, g/dL	3.98±0.24	4.69±0.21	.036
Pleural effusion LDH level, U/L	267.7±59.4	211.7±35.6	.393
Pleural effusion sugar level, mg/dL	116.7±21.9	126.9±22.4	.765
Pleural effusion specific gravity	1.034±0.005	1.033±0.001	.685
Positive sputum AFB smear, n (%)	2 (10.0)	5 (14.3)	1.000
Positive sputum *M. tuberculosis* culture, n (%)	3 (15.0)	13 (38.1)	.124
Positive pleural effusion AFB smear, n (%)	1 (5.0)	3 (8.6)	1.000
Positive pleural effusion *M. tuberculosis* culture	5 (25.0)	12 (34.3)	.555

Abbreviations: AMTD, Amplified Mycobacterium Tuberculosis Direct; WBC, white blood cell count; LDH, *lactate dehydrogenase;* AFB, Acid fast bacilli.

### Etiology of pleural effusion with positive AMTD results

The etiologies of pleural effusion with positive AMTD results included TB pleurisy (57.1%, 20/35), malignant pleural effusion (17.1%, 6/35), para-pneumonic effusion (2.9%, 1/35), and connective tissue disease (5.7%, 2/35) (Table 5). Six patients (17.1%) had pleural effusion that was disappeared without anti-TB treatment or other specific therapy. These patients possibly had viral or partially treated bacterial infection. Their medical records were followed for 2–7 years and none of them developed tuberculosis. Therefore, they were categorized as non-TB cases of unknown etiology.

**Table 5:** The etiology of pleural effusion with positive AMTD results.

Diagnosis	Positive AMTD (n = 35)
Tuberculous pleurisy, n (%)	20 (57.1%)
Malignant pleural effusion, n (%)	6 (17.1%)
Para-pneumonic effusion, n (%)	1 (2.9%)
Connective tissue disease, n (%)	2 (5.7%)
Unknown etiology, n (%)	6 (17.1%)

Abbreviations: AMTD, Amplified Mycobacterium Tuberculosis Direct.

## Discussion

The present study demonstrates that AMTD tests are positive in about one-third of pleural effusion from patients with TB pleurisy. To provide clinical evidence for the rapid diagnosis of TB pleurisy, adding the AMTD test to the combination of sputum AFB smear, pleural effusion AFB smear, and pleural biopsy increases the diagnostic yield from 55% to 82.5%. Patients with symptom onset <18 days prior to pleural study have higher positive rates of AMTD than those without. However, there are false positive AMTD tests in the pleural effusion of patients with diseases other than TB pleurisy.

Conventional diagnostic tests for pleural TB include microscopic examination of the pleural fluid for AFB, mycobacterial culture of pleural fluid, sputum or pleural tissue, and pathology examination of pleural tissue for granulomatous inflammation. These tests have recognized limitations in clinical use, although in combination, they have been recognized as the best reference standard for evaluating the accuracy of novel tests [7]. Due to the time-consuming constraint of mycobacterial culture, only sputum AFB smear, pleural effusion AFB smear, and pleural biopsy can provide clinical evidence for the early diagnosis of pleural TB. Rapid diagnosis and treatment have long been considered a crucial factor for better outcome of TB patients. A delay in anti-TB treatment of more than 14 days has been associated with worse clinical outcome of pleural TB patients [6]. In the present study, sputum AFB smear, pleural effusion AFB smear, and pleural biopsy offer a rapid evidence for TB pleurisy in 55% of patients. At the same time, the diagnostic yield is increased to 82.5% by combining the AMTD test with these conventional methods. However, due to the presence of false positive result in AMTD test, the specificity fell to 74% when combining AMTD to these conventional methods. The high diagnostic yield of combined AMTD test with conventional methods for the early diagnosis of TB pleurisy may therefore prevent delays in the administration of anti-TB treatment or the need for further invasive procedures like CT-guided or surgical biopsy to reach a definitive diagnosis.

NAATs have improved the accuracy and speed of diagnosing tuberculosis based on the examination of respiratory specimens [18]. For pleural effusion specimens, however, previous studies have shown highly variable results regarding the usefulness of NAATs [19]. According to previous reports, the sensitivity of AMTD test in pleural effusion for diagnosis of TB pleural effusion ranged from 20% to 100%. The sensitivity of 36.4% in our study was in line with previous reports. Recent evidence suggest that the sensitivity of *M. tuberculosis* AMTD testing is largely dependent on the bacillary load of the specimens [15]. The present study has disclosed that patients with positive AMTD test have higher percentages of neutrophils in pleural effusion compared with those with negative results. In pleural TB, previous studies have shown that neutrophils are the predominant cells in the pleural cavity, followed by a subsequent change to lymphocytic predominance [6]. The higher percentage of neutrophils in patients with positive AMTD test suggests that these patients are relatively in the early course of pleural TB. Compatible with this result, the study disclosed that patients with symptom onset <18 days prior to pleural effusion study had a higher chance of positive AMTD study of pleural effusion than those received the test with longer duration of symptom.

In patients with positive *M. tuberculosis* in pleural effusion, only 5 of them had positive AMTD test. The low sensitivity of AMTD in pleural TB patients with positive *M. tuberculosis* culture may be caused by the presence of inhibitory substance in pleural effusion [20]. In addition, this study has false positive results of AMTD tests in patients with diagnosis other than pleural TB. These patients with false positive AMTD had been followed for 2–7 years and none of them develop tuberculosis. These false-positive results may have originated from cross-contamination during specimen processing, from cross-reaction with non-mycobacterial DNA, or from latent infection with tuberculosis [15], [21]. A previous report has suggested that the lung tissue of persons residing in an *M. tuberculosis* endemic area may be colonized with small amounts of *M. tuberculosis* without active infection detectable by PCR testing [22]. The high prevalence of tuberculosis in Taiwan may explain the false positive *M. tuberculosis* AMTD results in this study. Moreover, positive results in malignant pleural effusion may be due to temporary colonization or persistence of mycobacteria [23]. Compatible with previous reports, this study reveals that malignant pleural effusion is the most common cause of false positive results for AMTD tests. As such, positive *M. tuberculosis* AMTD results should be carefully interpreted in conjunction with clinical information, mycobacterial study results, and findings of pathologic examination of pleural biopsy specimens.

The major limitations of the present study are its retrospective character, which may lead to an underestimation of the incidence of TB pleurisy and bias in patient selection. Second, the sample size of the study is small; therefore, the results of the study should be interpreted with caution. A prospective study with larger sample size is warranted to further confirm the use of AMTD in diagnosis of TB pleural effusion. Lastly, the study did not measure adenosine deaminase (ADA) in pleural fluid. Nonetheless, the use of these makers may be limited in terms of availability in developing countries. Given the high sensitivity and specificity of ADA in diagnosis of TB pleurisy [24], [25], if ADA was used as a reference standard the results of the study may be affected. Therefore, the results of the study may only be applied in certain countries.

In conclusion, AMTD test provides a rapid result and have a potential role in diagnosis of TB pleurisy. Patients in the early course of TB pleurisy may have higher positive rate of positive AMTD tests. Due to the presence of false positive results, AMTD tests should be interpreted in conjunction with clinical information and other conventional diagnostic methods for pleural TB.
